# Some Similarity Measures of Neutrosophic Sets Based on the Euclidean Distance and Their Application in Medical Diagnosis

**DOI:** 10.1155/2018/7325938

**Published:** 2018-11-28

**Authors:** Donghai Liu, Guangyan Liu, Zaiming Liu

**Affiliations:** ^1^Department of Mathematics, Hunan University of Science and Technology, Xiangtan, Hunan 411201, China; ^2^Department of Mathematics, Central South University, Changsha, Hunan 410075, China

## Abstract

Similarity measure is an important tool in multiple criteria decision-making problems, which can be used to measure the difference between the alternatives. In this paper, some new similarity measures of single-valued neutrosophic sets (SVNSs) and interval-valued neutrosophic sets (IVNSs) are defined based on the Euclidean distance measure, respectively, and the proposed similarity measures satisfy the axiom of the similarity measure. Furthermore, we apply the proposed similarity measures to medical diagnosis decision problem; the numerical example is used to illustrate the feasibility and effectiveness of the proposed similarity measures of SVNSs and IVNSs, which are then compared to other existing similarity measures.

## 1. Introduction

The concept of fuzzy set (FS) *A*={〈*x*_*i*_, *u*_*A*_(*x*_*i*_)〉|*x*_*i*_ ∈ *X*} in *X*={*x*_1_, *x*_2_, …, *x*_*n*_} was proposed by Zadeh [1], where the membership degree *u*_*A*_(*x*_*i*_) is a single value between zero and one. The FS has been widely applied in many fields, such as medical diagnosis, image processing, supply decision-making [2–4], and so on. In some uncertain decision-making problems, the degree of membership is assumed not exactly as a numerical value but as an interval. Hence, Zadeh [5] proposed the interval-valued fuzzy set (IVFS). However, the FS and IVFS only have the membership degree, and they cannot describe the nonmembership degree of the element belonging to the set. For example, in the national entrance examination for postgraduate, a panel of ten professors evaluated the admission of a student; five professors considered the student can be accepted, three professors disapproved of his or her admission, and two professors remained neutral. In this case, the FS and IVFS cannot represent such information. In order to solve this problem, Atanassov et al. [6] proposed the intuitionistic fuzzy set (IFS) *E*={〈*x*_*i*_, *u*_*E*_(*x*_*i*_), *v*_*E*_(*x*_*i*_)〉|*x*_*i*_ ∈ *X*}, where *u*_*E*_(*x*_*i*_)(0 ≤ *u*_*E*_(*x*_*i*_) ≤ 1) and *v*_*E*_(*x*_*i*_)(0 ≤ *v*_*E*_(*x*_*i*_) ≤ 1) represent the membership degree and nonmembership degree, respectively, and the indeterminacy-membership degree *π*_*E*_(*x*_*i*_)=1 − *u*_*E*_(*x*_*i*_) − *v*_*E*_(*x*_*i*_). The IFS is more effective to deal with the vague information than the FS and IVFS. Then, the information about the admission of the student can be represented as an IFS *E*=0.5, 0.3, 0.2, where 0.5, 0.3,  and  0.2 stand for the membership degree, nonmembership degree, and indeterminacy-membership degree, respectively. However, the IFS also have limitation in expressing the decision information. For example, three groups of experts evaluate the benefits of the stock, a group of experts thinks the possibility of the stock that will be profitable is 0.6, the second group of experts thinks the possibility of loss is 0.3, the third group of experts is not sure whether the stock that will be profitable is 0.4. In this case, the IFS cannot express such information because 0.6+0.3+0.4 > 1. Therefore, Wang et al. [7] proposed a single-valued neutrosophic set (SVNS) *N*={〈*x*_*i*_, *T*_*N*_(*x*_*i*_), *I*_*N*_(*x*_*i*_), *F*_*N*_(*x*_*i*_)〉|*x*_*i*_ ∈ *X*}, where *T*_*N*_(*x*_*i*_), *I*_*N*_(*x*_*i*_)  and  *F*_*N*_(*x*_*i*_) represent the degree of the truth-membership, indeterminacy-membership, and falsity-membership, respectively, and they belong to [0,1]. So, the information about the benefits of the stock can be represented as *N*=0.6, 0.4, 0.3. However, due to the uncertainty of the decision-making environment in multiple criteria decision-making problems, the single numerical value cannot meet the needs of evaluating information. Then, Wang [8] defined the interval-valued neutrosophic set (IVNS) based on the SVNS, which used the interval to describe truth membership degree, indeterminacy membership degree, and falsity membership degree, respectively. Since the neutrosophic set was proposed, there have been some researchers focusing on this subject [9–12].

On the other hand, similarity measure is an important tool in multiple criteria decision-making problems, which can be used to measure the difference between the alternatives. Many studies about the similarity measure are obtained. For example, Beg et al. [13] proposed a similarity measure of FSs based on the concept of ϵ − fuzzy transitivity and discussed the degree of transitivity of different similarity measures. Song et al. [14] considered the similarity measure of IFSs and proposed corresponding distance measure between intuitionistic fuzzy belief functions. Majumdar and Samanta [15] proposed a similarity measure between SVNSs based on the membership degree.

In addition, cosine similarity measure is also an important similarity measure, and it can be defined as the inner product of two vectors divided by the product of their lengths. There are some scholars who study the cosine similarity measures [16–21]. For example, Ye [16] proposed the cosine similarity measure and weighted cosine similarity measure of IVFSs with risk preference, and they were applied to the supplier selection problem. Then, Ye [17] proposed the cosine similarity measure of IFSs and applied it to medical diagnosis and pattern recognition. Furthermore, Ye [18] defined the cosine similarity measure of SVNSs and IVNSs, but when the SVNSs *N*_1_ ≠ *N*_2_, cos(*N*_1_, *N*_2_)=1 (the example can be seen in Section 3). Furthermore, Ye [19] proposed the improved cosine similarity measures of SVNSs and IVNSs based on cosine function.

In this paper, we propose a new method to construct the similarity measures of SVNSs, which is based on the existing similarity measure proposed by Majumdar and Samanta [15] and Ye [18], respectively. They play an important role in practical application, especially in pattern recognition, medical diagnosis, and so on. Furthermore, we will propose the corresponding similarity measures of IVNSs.

The rest of the paper is organized as follows. In Section 2, the basic definition and some properties about SVNS and IVNS are given. In Section 3, we proposed a method to construct the new similarity measures of SVNSs and IVNSs, respectively. In Section 4, we apply the proposed new similarity measures to medical diagnosis problems, the numerical examples are used to illustrate the feasibility and effectiveness of the proposed similarity measures, which are then compared to other existing similarity measures. Finally, the conclusions and future studies are discussed in Section 5.

## 2. Preliminaries

In this section, we give some basic knowledge about the SVNS and the IVNS. Some existing distance measures are also introduced, which will be used in the next section.

### 2.1. SVNS

**Definition 1 .**

Given a fixed set *X*={*x*_1_, *x*_2_  ,…, *x*_*n*_} [7], the SVNS *N* in *X* is defined as follows:

(1) $$\begin{array}{c} N=\left\{\langle x_{i},T_{N}\left(x_{i}\right),I_{N}\left(x_{i}\right),F_{N}\left(x_{i}\right)\rangle \left|x_{i}\in X\right.\right\}, \end{array}$$

where the function *T*_*N*_(*x*_*i*_) : *X*⟶[0,1] defines the truth-membership degree, the function *I*_*N*_(*x*_*i*_) : *X*⟶[0,1] defines indeterminacy-membership degree, and the function *F*_*N*_(*x*_*i*_) : *X*⟶[0,1] defines the falsity-membership degree, respectively. For any SVNS *N*, it holds that 0 ≤ *T*_*N*_(*x*_*i*_)+*I*_*N*_(*x*_*i*_)+*F*_*N*_(*x*_*i*_) ≤ 3(∀*x*_*i*_ ∈ *X*).

For any two SVNSs *N*_1_={〈*x*_*i*_, *T*_*N*_1__(*x*_*i*_), *I*_*N*_1__(*x*_*i*_), *F*_*N*_1__(*x*_*i*_)〉|*x*_*i*_ ∈ *X*} and *N*_2_={〈*x*_*i*_, *T*_*N*_2__(*x*_*i*_), *I*_*N*_2__(*x*_*i*_), *F*_*N*_2__(*x*_*i*_)〉|*x*_*i*_∈*X*}}, the following properties are satisfied:

1. *N*_1_⊆*N*_2_ if and only if *T*_*N*_1__(*x*_*i*_) ≤ *T*_*N*_2__(*x*_*i*_), *I*_*N*_1__(*x*_*i*_) ≥ *I*_*N*_2__(*x*_*i*_), and *F*_*N*_1__(*x*_*i*_) ≥ *F*_*N*_2__(*x*_*i*_)
2. *N*_1_=*N*_2_ if and only if *N*_1_⊆*N*_2_ and *N*_2_⊆*N*_1_

### 2.2. IVNS

**Definition 2 .**

Given a fixed set X={*x*_1_, *x*_2_,…, *x*_*n*_} [8], the IVNS *N*′ on *X* is defined as follows:

(2) $$\begin{array}{c} N^{\prime }=\left\{\langle x_{i},\left[T_{N^{\prime }}^{L}\left(x_{i}\right),T_{N^{\prime }}^{U}\left(x_{i}\right)\right],\left[I_{N^{\prime }}^{L}\left(x_{i}\right),I_{N^{\prime }}^{U}\left(x_{i}\right)\right],\left[F_{N^{\prime }}^{L}\left(x_{i}\right),F_{N^{\prime }}^{U}\left(x_{i}\right)\right]\rangle \left|x_{i}\in X\right.\right\}, \end{array}$$

where *T*_*N*′_(*x*_*i*_)=[*T*_*N*′_^*L*^(*x*_*i*_), *T*_*N*′_^*U*^(*x*_*i*_)], *I*_*N*′_(*x*_*i*_)=[*I*_*N*′_^*L*^(*x*_*i*_), *I*_*N*′_^*U*^(*x*_*i*_)], and *F*_*N*′_(*x*_*i*_)=[*F*_*N*′_^*L*^(*x*_*i*_), *F*_*N*′_^*U*^(*x*_*i*_)] represent the truth-membership function, the indeterminacy-membership function, and the falsity-membership function, respectively. For any *x*_*i*_ ∈ *X*, it holds that *T*_*N*′_(*x*_*i*_), *I*_*N*′_(*x*_*i*_), *F*_*N*′_(*x*_*i*_)⊆[0,1] and 0 ≤ *T*_*N*′_^*U*^(*x*_*i*_)+*I*_*N*′_^*U*^(*x*_*i*_)+*F*_*N*′_^*U*^(*x*_*i*_) ≤ 3.

For any two IVNSs *N*_1_′={〈*x*_*i*_, [*T*_*N*_1_′_^*L*^(*x*_*i*_), *T*_*N*_1_′_^*U*^(*x*_*i*_)], [*I*_*N*_1_′_^*L*^(*x*_*i*_), *I*_*N*_1_′_^*U*^(*x*_*i*_)], [*F*_*N*_1_′_^*L*^(*x*_*i*_), *F*_*N*_1_′_^*U*^(*x*_*i*_)]〉|*x*_*i*_ ∈ *X*} and *N*_2_′={〈*x*_*i*_, [*T*_*N*_2_′_^*L*^(*x*_*i*_), *T*_*N*_2_′_^*U*^(*x*_*i*_)], [*I*_*N*_2_′_^*L*^(*x*_*i*_), *I*_*N*_2_′_^*U*^(*x*_*i*_)], [*F*_*N*_2_′_^*L*^(*x*_*i*_), *F*_*N*_2_′_^*U*^(*x*_*i*_)]〉|*x*_*i*_ ∈ *X*}, the following properties are satisfied:

1. *N*_1_′⊆*N*_2_′ if and only if *T*_*N*_1_′_^*L*^(*x*_*i*_) ≤ *T*_*N*_2_′_^*L*^(*x*_*i*_), *T*_*N*_1_′_^*U*^(*x*_*i*_) ≤ *T*_*N*_2_′_^*U*^(*x*_*i*_), *I*_*N*_1_′_^*L*^(*x*_*i*_) ≥ *I*_*N*_2_′_^*L*^(*x*_*i*_), *I*_*N*_1_′_^*U*^(*x*_*i*_) ≥ *I*_*N*_2_′_^*U*^(*x*_*i*_), *F*_*N*_1_′_^*L*^(*x*_*i*_) ≥ *F*_*N*_2_′_^*L*^(*x*_*i*_), and*F*_*N*_1_′_^*U*^(*x*_*i*_) ≥ *F*_*N*_2_′_^*U*^(*x*_*i*_)
2. *N*_1_′=*N*_2_′ if and only if *N*_1_′⊆*N*_2_′ and *N*_2_′⊆*N*_1_′

**Remark 1 .**

When *T*_*N*_1_′_^*L*^(*x*_*i*_)=*T*_*N*_1_′_^*U*^(*x*_*i*_), *I*_*N*_1_′_^*L*^(*x*_*i*_)=*I*_*N*_1_′_^*U*^(*x*_*i*_), *F*_*N*_1_′_^*L*^(*x*_*i*_)=*F*_*N*_1_′_^*U*^(*x*_*i*_), the IVNS *N*_1_′ is reduced to the SVNS *N*_1_.

### 2.3. Existing Distance Measures between SVNSs and IVNSs

**Definition 3 .**

Let *N*_1_={〈*x*_*i*_, *T*_*N*_1__(*x*_*i*_), *I*_*N*_1__(*x*_*i*_), *F*_*N*_1__(*x*_*i*_)〉|*x*_*i*_ ∈ *X*} and *N*_2_={〈*x*_*i*_, *T*_*N*_2__(*x*_*i*_), *I*_*N*_2__(*x*_*i*_), *F*_*N*_2__(*x*_*i*_)〉|*x*_*i*_∈*X*}} be any two SVNSs in *X*={*x*_1_, *x*_2_, …, *x*_*n*_} [15]; then, the Euclidean distance between SVNSs *N*_1_ and *N*_2_ is defined as follows:

(3) $$\begin{array}{c} D_{\text{SVNS}}\left(N_{1},N_{2}\right)=\sqrt{\frac{\sum_{i=1}^{n}\left[{\left(T_{N_{1}}\left(x_{i}\right)-T_{N_{2}}\left(x_{i}\right)\right)}^{2}+{\left(I_{N_{1}}\left(x_{i}\right)-I_{N_{2}}\left(x_{i}\right)\right)}^{2}+{\left(F_{N_{1}}\left(x_{i}\right)-F_{N_{2}}\left(x_{i}\right)\right)}^{2}\right]}{3n}}. \end{array}$$

**Definition 4 .**

Let *N*_1_′={〈*x*_*i*_, [*T*_*N*_1_′_^*L*^(*x*_*i*_), *T*_*N*_1_′_^*U*^(*x*_*i*_)], [*I*_*N*_1_′_^*L*^(*x*_*i*_), *I*_*N*_1_′_^*U*^(*x*_*i*_)], [*F*_*N*_1_′_^*L*^(*x*_*i*_), *F*_*N*_1_′_^*U*^(*x*_*i*_)]〉|*x*_*i*_ ∈ *X*} and *N*_2_′={〈*x*_*i*_, [*T*_*N*_2_′_^*L*^(*x*_*i*_), *T*_*N*_2_′_^*U*^(*x*_*i*_)], [*I*_*N*_2_′_^*L*^(*x*_*i*_), *I*_*N*_2_′_^*U*^(*x*_*i*_)], [*F*_*N*_2_′_^*L*^(*x*_*i*_), *F*_*N*_2_′_^*U*^(*x*_*i*_)]〉|*x*_*i*_ ∈ *X*} be any two IVNSs in *X*={*x*_1_, *x*_2_, …, *x*_*n*_} [22]; the Euclidean distance between IVNSs *N*_1_′ and *N*_2_′ is defined as follows:

(4) $$\begin{array}{c} D_{\text{IVNS}}\left(N_{1}^{\prime },N_{2}^{\prime }\right)=\sqrt{\frac{\sum_{i=1}^{n}\left[{\left(T_{N_{1}^{\prime }}^{L}\left(x_{i}\right)-T_{N_{2}^{\prime }}^{L}\left(x_{i}\right)\right)}^{2}+{\left(T_{N_{1}^{\prime }}^{U}\left(x_{i}\right)-T_{N_{2}^{\prime }}^{U}\left(x_{i}\right)\right)}^{2}+{\left(I_{N_{1}^{\prime }}^{L}\left(x_{i}\right)-I_{N_{2}^{\prime }}^{L}\left(x_{i}\right)\right)}^{2}+{\left(I_{N_{1}^{\prime }}^{U}\left(x_{i}\right)-I_{N_{2}^{\prime }}^{U}\left(x_{i}\right)\right)}^{2}+{\left(F_{N_{1}^{\prime }}^{L}\left(x_{i}\right)-F_{N_{2}^{\prime }}^{L}\left(x_{i}\right)\right)}^{2}+{\left(F_{N_{1}^{\prime }}^{U}\left(x_{i}\right)-F_{N_{2}^{\prime }}^{U}\left(x_{i}\right)\right)}^{2}\right]}{6n}}. \end{array}$$

Next, we propose a new method to construct the similarity measures of SVNSs and IVNSs based on the Euclidean distance measure.

## 3. Several New Similarity Measures

The similarity measure is a most widely used tool to evaluate the relationship between two sets. The following axiom about the similarity measure of SVNSs (or IVNSs) should be satisfied:

**Lemma 1 .**

Let *X*={*x*_1_, *x*_2_, …, *x*_*n*_} be the universal set [18] if the similarity measure S(*N*_1_, *N*_2_) between SVNSs (or IVNSs) *N*_1_ and *N*_2_ satisfies the following properties:

1. 0 ≤ *S*(*N*_1_, *N*_2_) ≤ 1
2. *S*(*N*_1_, *N*_2_)=1 if and only if *N*_1_=*N*_2_
3. *S*(*N*_1_, *N*_2_)=*S*(*N*_2_, *N*_1_).

Then, the similarity measure S(*N*_1_, *N*_2_) is a genuine similarity measure.

### 3.1. The New Similarity Measures between SVNSs

To introduce the new similarity measure between SVNSs, we first review the similarity measure *S*_1SVNS_ between *N*_1_ and *N*_2_ defined by Majumdar et al. [15], which is given as follows:

**Definition 5 .**

Let *X*={*x*_1_, *x*_2_,…, *x*_*n*_} be a universal set [15], for any two SVNSs *N*_1_={〈*x*_*i*_, *T*_*N*_1__(*x*_*i*_), *I*_*N*_1__(*x*_*i*_), *F*_*N*_1__(*x*_*i*_)〉|*x*_*i*_ ∈ *X*} and *N*_2_={〈*x*_*i*_, *T*_*N*_2__(*x*_*i*_), *I*_*N*_2__(*x*_*i*_), *F*_*N*_2__(*x*_*i*_)〉|*x*_*i*_∈*X*}; the similarity measure of SVNSs between *N*_1_ and *N*_2_ is defined as follows:

(5) $$\begin{array}{c} S_{1\text{SVNS}}\left(N_{1},N_{2}\right)=\frac{\sum_{i=1}^{n}\left(\min\left(T_{N_{1}}\left(x_{i}\right),T_{N_{2}}\left(x_{i}\right)\right)+\min\left(I_{N_{1}}\left(x_{i}\right),I_{N_{2}}\left(x_{i}\right)\right)+\min\left(F_{N_{1}}\left(x_{i}\right),F_{N_{2}}\left(x_{i}\right)\right)\right)}{\sum_{i=1}^{n}\left(\max\left(T_{N_{1}}\left(x_{i}\right),T_{N_{2}}\left(x_{i}\right)\right)+\max\left(I_{N_{1}}\left(x_{i}\right),I_{N_{2}}\left(x_{i}\right)\right)+\max\left(F_{N_{1}}\left(x_{i}\right),F_{N_{2}}\left(x_{i}\right)\right)\right)}. \end{array}$$

It is already known that the similarity measure *S*_1SVNS_ defined by Majumdar et al. [15] satisfies the properties in Lemma 1. It is proposed based on the membership degree; in this section, we adopt the various methods for calculating the similarity measure between neutrosophic sets.

Firstly, we propose a new method to construct a new similarity measure of SVNSs, which is based on the similarity measure proposed by Majumdar et al. [15] and the Euclidean distance; it can be defined as follows:

**Definition 6 .**

Let *X*={*x*_1_, *x*_2_,…, *x*_*n*_} be a universal set, for any two SVNSs *N*_1_={*x*_*i*_, *T*_*N*_1__(*x*_*i*_), *I*_*N*_1__(*x*_*i*_), *F*_*N*_1__(*x*_*i*_)|*x*_*i*_ ∈ *X*} and *N*_2_={*x*_*i*_, *T*_*N*_2__(*x*_*i*_), *I*_*N*_2__(*x*_*i*_), *F*_*N*_2__(*x*_*i*_)|*x*_*i*_∈*X*}; a new similarity measure *S*_1SVNS_^*∗*^(*N*_1_, *N*_2_) is defined as follows:

(6) $$\begin{array}{c} S_{1\text{SVNS}}^{*}\left(N_{1},N_{2}\right)=\frac{1}{2}\left(S_{1\text{SVNS}}\left(N_{1},N_{2}\right)+1-D_{\text{SVNS}}\left(N_{1},N_{2}\right)\right). \end{array}$$

The proposed similarity measure of SVNSs satisfies the following Theorem 1.

**Theorem 1 .**

The similarity measure *S*_1SVNS_^*∗*^(*N*_1_, *N*_2_) between *N*_1_={〈*x*_*i*_, *T*_*N*_1__(*x*_*i*_), *I*_*N*_1__(*x*_*i*_), *F*_*N*_1__(*x*_*i*_)〉|*x*_*i*_ ∈ *X*} and *N*_2_={〈*x*_*i*_, *T*_*N*_2__(*x*_*i*_), *I*_*N*_2__(*x*_*i*_), *F*_*N*_2__(*x*_*i*_)〉|*x*_*i*_∈*X*} satisfies the following properties:

1. 0 ≤ *S*_1SVNS_^*∗*^(*N*_1_, *N*_2_) ≤ 1
2. *S*_1SVNS_^*∗*^(*N*_1_, *N*_2_)=1 if and only if *N*_1_=*N*_2_
3. *S*_1SVNS_^*∗*^(*N*_1_, *N*_2_)=*S*_1SVNS_^*∗*^(*N*_2_, *N*_1_)

**Proof**

1. Because *D*_SVNS_(*N*_1_, *N*_2_) is an Euclidean distance measure, obviously, 0 ≤ *D*_SVNS_(*N*_1_, *N*_2_) ≤ 1. Furthermore, according to Proposition 4.2.2 by Majumdar et al. [15], we know 0 ≤ *S*_1SVNS_(*N*_1_, *N*_2_) ≤ 1. Then, 0 ≤ 1/2(*S*_1SVNS_(*N*_1_, *N*_2_)+1 − *D*_SVNS_(*N*_1_, *N*_2_)) ≤ 1, i.e., 0 ≤ *S*_1SVNS_^*∗*^(*N*_1_, *N*_2_) ≤ 1.
2. If *S*_1SVNS_^*∗*^(*N*_1_, *N*_2_)=1, we have *S*_1SVNS_(*N*_1_, *N*_2_)+1 − *D*_SVNS_(*N*_1_, *N*_2_)=2, that is, *S*_1SVNS_(*N*_1_, *N*_2_)=1+*D*_SVNS_(*N*_1_, *N*_2_). Because *D*_SVNS_(*N*_1_, *N*_2_) is the Euclidean distance measure, 0 ≤ *D*_SVNS_(*N*_1_, *N*_2_) ≤ 1. Furthermore, 0 ≤ *S*_1SVNS_(*N*_1_, *N*_2_) ≤ 1 is obtained in Proposition 4.2.2 [15], then *S*_1SVNS_(*N*_1_, *N*_2_)=1 and *D*_SVNS_(*N*_1_, *N*_2_)=0 should be established at the same time. If the Euclidean distance measure *D*_SVNS_(*N*_1_, *N*_2_)=0, *N*_1_=*N*_2_ is obvious. According to Proposition 4.2.2 by Majumdar et al. [15], when *S*_1SVNS_(*N*_1_, *N*_2_)=1, *N*_1_=*N*_2_; so, if *S*_1SVNS_^*∗*^(*N*_1_, *N*_2_)=1, *N*_1_=*N*_2_ is obtained.

On the other hand, when *N*_1_=*N*_2_, according to formulae (3) and (5) *D*_SVNS_(*N*_1_, *N*_2_)=0 and *S*_1SVNS_(*N*_1_, *N*_2_)=1 are obtained respectively. Furthermore, we can get *S*_1SVNS_^*∗*^(*N*_1_, *N*_2_)=1.

(3) *S*_1SVNS_^*∗*^(*N*_1_, *N*_2_)=*S*_1SVNS_^*∗*^(*N*_2_, *N*_1_) is straightforward.

From Theorem 1, we know the proposed new similarity measure *S*_1SVNS_^*∗*^(*N*_1_, *N*_2_) is a genuine similarity measure.

On the other hand, cosine similarity measure is also an important similarity measure. In 2014, Ye [18] proposed a cosine similarity measure between SVNSs as follows:

**Definition 7 .**

Let X={*x*_1_, *x*_2_, …, *x*_*n*_} be a universal set [18], for any two SVNSs *N*_1_={〈*x*_*i*_, *T*_*N*_1__(*x*_*i*_), *I*_*N*_1__(*x*_*i*_), *F*_*N*_1__(*x*_*i*_)〉|*x*_*i*_ ∈ *X*} and *N*_2_={〈*x*_*i*_, *T*_*N*_2__(*x*_*i*_), *I*_*N*_2__(*x*_*i*_), *F*_*N*_2__(*x*_*i*_)〉|*x*_*i*_ ∈ *X*}, the cosine similarity measure between *N*_1_ and *N*_2_ is defined as follows:

(7) $$\begin{array}{c} S_{2\text{SVNS}}\left(N_{1},N_{2}\right)=\frac{1}{n}\overset{n}{\underset{i=1}{\sum }}\frac{T_{N_{1}}\left(x_{i}\right)T_{N_{2}}\left(x_{i}\right)+I_{N_{1}}\left(x_{i}\right)I_{N_{2}}\left(x_{i}\right)+F_{N_{1}}\left(x_{i}\right)F_{N_{2}}\left(x_{i}\right)}{\sqrt{T_{N_{1}}^{2}\left(x_{i}\right)+I_{N_{1}}^{2}\left(x_{i}\right)+F_{N_{1}}^{2}\left(x_{i}\right)}\sqrt{T_{N_{2}}^{2}\left(x_{i}\right)+I_{N_{2}}^{2}\left(x_{i}\right)+F_{N_{2}}^{2}\left(x_{i}\right)}}. \end{array}$$

From Example 1, we know the cosine similarity measure defined by Ye [18] does not satisfy Lemma 1.

**Example 1 .**

For two SVNSs *N*_1_=*x*, 0.4, 0.2, 0.6 and *N*_2_=*x*, 0.2, 0.1, 0.3, we can easily know *N*_1_ ≠ *N*_2_. But using formula (7) to calculate the cosine similarity measure *S*_2SVNS_(*N*_1_, *N*_2_), we have *S*_2SVNS_(*N*_1_, *N*_2_)=1. That is to say, when *N*_1_ ≠ *N*_2_, *S*_2SVNS_(*N*_1_, *N*_2_)=1, which means the cosine similarity measure *S*_2SVNS_(*N*_1_, *N*_2_) defined by Ye [18] does not satisfy the necessary condition of property 2 in Lemma 1; thus, it is not a genuine similarity measure. Furthermore, Ye [19] proposed the improved cosine similarity measures of SVNS based on the cosine similarity measure proposed by Ye [18], which overcomes its shortcoming.

In this paper, we go on proposing another new similarity measure of SVNSs based on the cosine similarity measure proposed by Ye [18] and the Euclidean distance *D*_SVNS_. It considers the similarity measure not only from the point of view of algebra but also from the point of view of geometry, which can be defined as:

**Definition 8 .**

Let *X*={*x*_1_, *x*_2_,…, *x*_*n*_} be a universal set, for any two SVNSs *N*_1_={〈*x*_*i*_, *T*_*N*_1__(*x*_*i*_), *I*_*N*_1__(*x*_*i*_), *F*_*N*_1__(*x*_*i*_)〉|*x*_*i*_ ∈ *X*} and *N*_2_={〈*x*_*i*_, *T*_*N*_2__(*x*_*i*_), *I*_*N*_2__(*x*_*i*_), *F*_*N*_2__(*x*_*i*_)〉|*x*_*i*_ ∈ *X*}; a new similarity measure *S*_2SVNS_^*∗*^(*N*_1_, *N*_2_) is defined as follows:

(8) $$\begin{array}{c} S_{2\text{SVNS}}^{*}\left(N_{1},N_{2}\right)=\frac{1}{2}\left(S_{2\text{SVNS}}\left(N_{1},N_{2}\right)+1-D_{\text{SVNS}}\left(N_{1},N_{2}\right)\right). \end{array}$$

**Remark 2 .**

Using formula (8) to calculate Example 1 again, for two SVNSs *N*_1_=*x*, 0.4, 0.2, 0.6 and *N*_2_=*x*, 0.2, 0.1, 0.3, we have *S*_2SVNS_^*∗*^(*N*_1_, *N*_2_)=0.8920. We can see that the proposed new similarity measure *S*_2SVNS_^*∗*^(*N*_1_, *N*_2_) overcomes the shortcoming of cosine similarity measure *S*_2SVNS_(*N*_1_, *N*_2_) defined by Ye [18].

**Theorem 2 .**

The similarity measure *S*_2SVNS_^*∗*^(*N*_1_, *N*_2_) between *N*_1_={〈*x*_*i*_, *T*_*N*_1__(*x*_*i*_), *I*_*N*_1__(*x*_*i*_), *F*_*N*_1__(*x*_*i*_)〉|*x*_*i*_ ∈ *X*} and *N*_2_={〈*x*_*i*_, *T*_*N*_2__(*x*_*i*_), *I*_*N*_2__(*x*_*i*_), *F*_*N*_2__(*x*_*i*_)〉|*x*_*i*_ ∈ *X*} satisfies the following properties:

1. 0 ≤ *S*_2SVNS_^*∗*^(*N*_1_, *N*_2_) ≤ 1
2. *S*_2SVNS_^*∗*^(*N*_1_, *N*_2_)=1 if and only if *N*_1_=*N*_2_
3. *S*_2SVNS_^*∗*^(*N*_1_, *N*_2_)=*S*_2SVNS_^*∗*^(*N*_2_, *N*_1_)

**Proof**

The proof of the properties (1) and (3) are similar to Theorem 1; here, we only give the proof of property (2).

If *S*_2SVNS_^*∗*^(*N*_1_, *N*_2_)=1, we have *S*_2SVNS_(*N*_1_, *N*_2_)+1 − *D*_SVNS_(*N*_1_, *N*_2_)=2, i.e., *S*_2SVNS_(*N*_1_, *N*_2_)=1+*D*_SVNS_(*N*_1_, *N*_2_). Because *D*_SVNS_(*N*_1_, *N*_2_) is the Euclidean distance measure, 0 ≤ *D*_SVNS_(*N*_1_, *N*_2_) ≤ 1. According to the property of *S*_2SVNS_(*N*_1_, *N*_2_) in Ye [18], 0 ≤ *S*_2SVNS_(*N*_1_, *N*_2_) ≤ 1; then, *S*_2SVNS_(*N*_1_, *N*_2_)=1 and *D*_SVNS_(*N*_1_, *N*_2_)=0 should be held at the same time. When *S*_2SVNS_(*N*_1_, *N*_2_)=1, we have *T*_*N*_1__(*x*_*i*_)=*k* · *T*_*N*_2__(*x*_*i*_), *I*_*N*_1__(*x*_*i*_)=*k* · *I*_*N*_2__(*x*_*i*_), and *F*_*N*_1__(*x*_*i*_)=*k* · *F*_*N*_2__(*x*_*i*_) (*k* is a constant). When *D*_SVNS_(*N*_1_, *N*_2_)=0, we have *N*_1_=*N*_2_. Then *N*_1_=*N*_2_ is obtained.

On the other hand, according to formulae (3) and (7), if *N*_1_=*N*_2_, *D*_SVNS_(*N*_1_, *N*_2_)=0 and *S*_2SVNS_(*N*_1_, *N*_2_)=1 are obtained, respectively; then we can get *S*_2SVNS_^*∗*^(*N*_1_, *N*_2_)=1.

Thus, *S*_2SVNS_^*∗*^(*N*_1_, *N*_2_) satisfies all the properties in Theorem 2.

### 3.2. Some New Similarity Measures between IVNSs

In some situations, it is difficult to provide the truth-membership degree, false-membership degree, and indeterminate-membership degree with a precise numerical value; Wang [8] used the interval numbers to express the related membership degrees. Furthermore, Broumi et al. [22] proposed the corresponding similarity measure of IVNSs based on the similarity measure *S*_1SVNS_ proposed by Majumdar et al. [15].

**Definition 9 .**

Let *X*={*x*_1_, *x*_2_,…, *x*_*n*_} be a universal set, for any two IVNSs *N*_1_′={〈*x*_*i*_, [*T*_*N*_1_′_^*L*^(*x*_*i*_), *T*_*N*_1_′_^*U*^(*x*_*i*_)], [*I*_*N*_1_′_^*L*^(*x*_*i*_), *I*_*N*_1_′_^*U*^(*x*_*i*_)], [*F*_*N*_1_′_^*L*^(*x*_*i*_), *F*_*N*_1_′_^*U*^(*x*_*i*_)]〉|*x*_*i*_ ∈ *X*} and *N*_2_′={〈*x*_*i*_, [*T*_*N*_2_′_^*L*^(*x*_*i*_)*T*_*N*_2_′_^*U*^(*x*_*i*_)], [*I*_*N*_2_′_^*L*^(*x*_*i*_), *I*_*N*_2_′_^*U*^(*x*_*i*_)], [*F*_*N*_2_′_^*L*^(*x*_*i*_), *F*_*N*_2_′_^*U*^(*x*_*i*_)]〉|*x*_*i*_ ∈ *X*} [22]; the similarity measure between IVNSs *N*_1_′ and *N*_2_′ is defined as follows:

(9) $$\begin{array}{c} S_{1\text{IVNS}}\left(N_{1}^{\prime },N_{2}^{\prime }\right)=\frac{\sum_{i=1}^{n}\left\{\min\left(T_{N_{1}^{\prime }}^{L}\left(x_{i}\right),T_{N_{2}^{\prime }}^{L}\left(x_{i}\right)\right)+\min\left(T_{N_{1}^{\prime }}^{U}\left(x_{i}\right),T_{N_{2}^{\prime }}^{U}\left(x_{i}\right)\right)+\min\left(I_{N_{1}^{\prime }}^{L}\left(x_{i}\right),I_{N_{2}^{\prime }}^{L}\left(x_{i}\right)\right)+\min\left(I_{N_{1}^{\prime }}^{U}\left(x_{i}\right),I_{N_{2}^{\prime }}^{U}\left(x_{i}\right)\right)+\min\left(F_{N_{1}^{\prime }}^{L}\left(x_{i}\right),F_{N_{2}^{\prime }}^{L}\left(x_{i}\right)\right)+\min\left(F_{N_{1}^{\prime }}^{U}\left(x_{i}\right),F_{N_{2}^{\prime }}^{U}\left(x_{i}\right)\right)\right\}}{\sum_{i=1}^{n}\left\{\max\left(T_{N_{1}^{\prime }}^{L}\left(x_{i}\right),T_{N_{2}^{\prime }}^{L}\left(x_{i}\right)\right)+\max\left(T_{N_{1}^{\prime }}^{U}\left(x_{i}\right),T_{N_{2}^{\prime }}^{U}\left(x_{i}\right)\right)+\max\left(I_{N_{1}^{\prime }}^{L}\left(x_{i}\right),I_{N_{2}^{\prime }}^{L}\left(x_{i}\right)\right)+\max\left(I_{N_{1}^{\prime }}^{U}\left(x_{i}\right),I_{N_{2}^{\prime }}^{U}\left(x_{i}\right)\right)+\max\left(F_{N_{1}^{\prime }}^{L}\left(x_{i}\right),F_{N_{2}^{\prime }}^{L}\left(x_{i}\right)\right)+\max\left(F_{N_{1}^{\prime }}^{U}\left(x_{i}\right),F_{N_{2}^{\prime }}^{U}\left(x_{i}\right)\right)\right\}}. \end{array}$$

**Remark 3 .**

If *T*_*N*_*j*_′_^*L*^(*x*_*i*_)=*T*_*N*_*j*_′_^*U*^(*x*_*i*_), *I*_*N*_*j*_′_^*L*^(*x*_*i*_)=*I*_*N*_*j*_′_^*U*^(*x*_*i*_), *F*_*N*_*j*_′_^*L*^(*x*_*i*_)=*F*_*N*_*j*_′_^*U*^(*x*_*i*_)(*j*=1,2), then the similarity measure *S*_1IVNS_(*N*_1_′, *N*_2_′) is reduced to the similarity measure *S*_1SVNS_(*N*_1_, *N*_2_).

Similarly to Section 3.1, we propose a corresponding similarity measure between IVNSs, which is based on the similarity measure *S*_1IVNS_(*N*_1_′, *N*_2_′) and the Euclidean distance *D*_IVNS_(*N*_1_′, *N*_2_′) defined in Definition 4.

***Definition 10*.**

Let X={*x*_1_, *x*_2_,…, *x*_*n*_} be a universal set, for any two IVNSs *N*_1_′={*x*_*i*_, [*T*_*N*_1_′_^*L*^(*x*_*i*_), *T*_*N*_1_′_^*U*^(*x*_*i*_)], [*I*_*N*_1_′_^*L*^(*x*_*i*_), *I*_*N*_1_′_^*U*^(*x*_*i*_)], [*F*_*N*_1_′_^*L*^(*x*_*i*_), *F*_*N*_1_′_^*U*^(*x*_*i*_)]|*x*_*i*_ ∈ *X*} and *N*_2_′={*x*_*i*_, [*T*_*N*_2_′_^*L*^}(*x*_*i*_), *T*_*N*_2_′_^*U*^(*x*_*i*_)], [*I*_*N*_2_′_^*L*^(*x*_*i*_), *I*_*N*_2_′_^*U*^(*x*_*i*_)], [*F*_*N*_2_′_^*L*^(*x*_*i*_), *F*_*N*_2_′_^*U*^(*x*_*i*_)]|*x*_*i*_ ∈ *X*}; a new similarity measure *S*_1IVNS_^*∗*^(*N*_1_′, *N*_2_′) is defined as follows:

(10) $$\begin{array}{c} S_{1\text{IVNS}}^{*}\left(N_{1}^{\prime },N_{2}^{\prime }\right)=\frac{1}{2}\left(S_{1\text{IVNS}}\left(N_{1}^{\prime },N_{2}^{\prime }\right)+1-D_{\text{IVNS}}\left(N_{1}^{\prime },N_{2}^{\prime }\right)\right). \end{array}$$

The proposed similarity measure also satisfies Theorem 3.

**Theorem 3 .**

*The similarity measureS*_1IVNS_^*∗*^(*N*_1_′, *N*_2_′)*satisfies the following properties:*

1. 0 ≤ *S*_1IVNS_^*∗*^(*N*_1_′, *N*_2_′) ≤ 1
2. *S*_1IVNS_^*∗*^(*N*_1_′, *N*_2_′)=1*if and only ifN*_1_′=*N*_2_′
3. *S*_1IVNS_^*∗*^(*N*_1_′, *N*_2_′)=*S*_1IVNS_^*∗*^(*N*_2_′, *N*_1_′)

**Proof**

The proof is similar to Theorem 1; hence, we omit it here.

Next, we will use the same method to define the similarity measure *S*_2IVNS_^*∗*^(*N*_1_′, *N*_2_′) between IVNS, which is based on the cosine similarity measure *S*_2IVNS_(*N*_1_′, *N*_2_′) proposed by Ye [21] (Definition 11) and the Euclidean distance *D*_IVNS_(*N*_1_′, *N*_2_′) defined in formula (4).

***Definition 11*.**

Let *X*={*x*_1_, *x*_2_,…, *x*_*n*_} be a universal set, for any two IVNSs *N*_1_′={〈*x*_*i*_, [*T*_*N*_1_′_^*L*^(*x*_*i*_), *T*_*N*_1_′_^*U*^(*x*_*i*_)], [*I*_*N*_1_′_^*L*^(*x*_*i*_), *I*_*N*_1_′_^*U*^(*x*_*i*_)], [*F*_*N*_1_′_^*L*^(*x*_*i*_), *F*_*N*_1_′_^*U*^(*x*_*i*_)]〉|*x*_*i*_ ∈ *X*} and *N*_2_′={〈*x*_*i*_, [*T*_*N*_2_′_^*L*^(*x*_*i*_), *T*_*N*_2_′_^*U*^(*x*_*i*_)], [*I*_*N*_2_′_^*L*^(*x*_*i*_), *I*_*N*_2_′_^*U*^(*x*_*i*_)], [*F*_*N*_2_′_^*L*^(*x*_*i*_), *F*_*N*_2_′_^*U*^(*x*_*i*_)]〉|*x*_*i*_ ∈ *X*}; the cosine similarity measure *S*_2IVNS_^*∗*^(*N*_1_′, *N*_2_′) is defined as follows [21]:

(11) $$\begin{array}{c} S_{2\text{IVNS}}\left(N_{1}^{\prime },N_{2}^{\prime }\right)=\frac{1}{n}\overset{n}{\underset{i=1}{\sum }}\frac{T_{N_{1}^{\prime }}^{L}\left(x_{i}\right)T_{N_{2}^{\prime }}^{L}\left(x_{i}\right)+T_{N_{1}^{\prime }}^{U}\left(x_{i}\right)T_{N_{2}^{\prime }}^{U}\left(x_{i}\right)+I_{N_{1}^{\prime }}^{L}\left(x_{i}\right)I_{N_{2}^{\prime }}^{L}\left(x_{i}\right)+I_{N_{1}^{\prime }}^{U}\left(x_{i}\right)I_{N_{2}^{\prime }}^{U}\left(x_{i}\right)+F_{N_{1}^{\prime }}^{L}\left(x_{i}\right)F_{N_{2}^{\prime }}^{L}\left(x_{i}\right)+F_{N_{1}^{\prime }}^{U}\left(x_{i}\right)F_{N_{2}^{\prime }}^{U}\left(x_{i}\right)}{\sqrt{{\left(T_{N_{1}^{\prime }}^{L}\left(x_{i}\right)\right)}^{2}+{\left(T_{N_{1}^{\prime }}^{U}\left(x_{i}\right)\right)}^{2}+{\left(I_{N_{1}^{\prime }}^{L}\left(x_{i}\right)\right)}^{2}+{\left(I_{N_{1}^{\prime }}^{U}\left(x_{i}\right)\right)}^{2}+{\left(F_{N_{1}^{\prime }}^{L}\left(x_{i}\right)\right)}^{2}+{\left(F_{N_{1}^{\prime }}^{U}\left(x_{i}\right)\right)}^{2}}\sqrt{{\left(T_{N_{2}^{\prime }}^{L}\left(x_{i}\right)\right)}^{2}+{\left(T_{N_{2}^{\prime }}^{U}\left(x_{i}\right)\right)}^{2}+{\left(I_{N_{2}^{\prime }}^{L}\left(x_{i}\right)\right)}^{2}+{\left(I_{N_{2}^{\prime }}^{U}\left(x_{i}\right)\right)}^{2}+{\left(F_{N_{2}^{\prime }}^{L}\left(x_{i}\right)\right)}^{2}+{\left(F_{N_{2}^{\prime }}^{U}\left(x_{i}\right)\right)}^{2}}}. \end{array}$$

**Example 2 .**

For two IVNSs *N*_1_′=*x*, [0.3, 0.4], [0.2, 0.3], [0.4, 0.5] and *N*_2_′=*x*, [0.6, 0.8], [0.4, 0.6], [0.8, 1], according to formula (11), we have *S*_2IVNS_(*N*_1_′, *N*_2_′)=1, but *N*_1_′ ≠ *N*_2_′. In this case, the necessary condition of (2) in Lemma 1 is not satisfied. Therefore, the cosine similarity measure *S*_2IVNS_(*N*_1_′, *N*_2_′) proposed by Ye [22] is not a genuine similarity measure. Motivated by this, we will propose a new similarity measure *S*_2IVNS_^*∗*^(*N*_1_′, *N*_2_′) based on *S*_2IVNS_(*N*_1_′, *N*_2_′) and the Euclidean distance measure *D*_IVNS_(*N*_1_′, *N*_2_′) as follows:

***Definition 12*.**

Let *X*={*x*_1_, *x*_2_,…, *x*_*n*_} be a universal set, for any two IVNSs *N*_1_′={〈*x*_*i*_, [*T*_*N*_1_′_^*L*^(*x*_*i*_), *T*_*N*_1_′_^*U*^(*x*_*i*_)], [*I*_*N*_1_′_^*L*^(*x*_*i*_), *I*_*N*_1_′_^*U*^(*x*_*i*_)], [*F*_*N*_1_′_^*L*^(*x*_*i*_), *F*_*N*_1_′_^*U*^(*x*_*i*_)]〉|*x*_*i*_ ∈ *X*} and *N*_2_′={〈*x*_*i*_, [*T*_*N*_2_′_^*L*^(*x*_*i*_), *T*_*N*_2_′_^*U*^(*x*_*i*_)], [*I*_*N*_2_′_^*L*^(*x*_*i*_), *I*_*N*_2_′_^*U*^(*x*_*i*_)], [*F*_*N*_2_′_^*L*^(*x*_*i*_), *F*_*N*_2_′_^*U*^(*x*_*i*_)]〉|*x*_*i*_ ∈ *X*}; a new similarity measure *S*_2IVNS_^*∗*^(*N*_1_′, *N*_2_′) can be defined as follows:

(12) $$\begin{array}{c} S_{2\text{IVNS}}^{*}\left(N_{1}^{\prime },N_{2}^{\prime }\right)=\frac{1}{2}\left(S_{2\text{IVNS}}\left(N_{1}^{\prime },N_{2}^{\prime }\right)+1-D_{\text{IVNS}}\left(N_{1}^{\prime },N_{2}^{\prime }\right)\right). \end{array}$$

**Remark 4 .**

In Example 2, when *N*_1_′ ≠ *N*_2_′, the similarity measure *S*_2IVNS_(*N*_1_′, *N*_2_′)=1, this is inconsistent with the real decision problems. But, using formula (12) to calculate it again, we have *S*_2IVNS_^*∗*^(*N*_1_′, *N*_2_′)=0.8185. Obviously, the proposed similarity measure *S*_2IVNS_^*∗*^(*N*_1_′, *N*_2_′) can rectify the existing cosine similarity measure *S*_2IVNS_(*N*_1_′, *N*_2_′) defined by Ye [22].

**Theorem 4 .**

The similarity measure *S*_2IVNS_^*∗*^(*N*_1_′, *N*_2_′) satisfies the following properties:

1. 0 ≤ *S*_2IVNS_^*∗*^(*N*_1_′, *N*_2_′) ≤ 1
2. *S*_2IVNS_^*∗*^(*N*_1_′, *N*_2_′)=1 if and only if *N*_1_′=*N*_2_′
3. *S*_2IVNS_^*∗*^(*N*_1_′, *N*_2_′)=*S*_2IVNS_^*∗*^(*N*_2_′, *N*_1_′)

**Proof**

The proof is similar to Theorem 2, we also omit it here.

In the next section, we will apply the proposed new similarity measures to medical diagnosis decision problem; numerical examples are also given to illustrate the application and effectiveness of the proposed new similarity measures.

## 4. Applications of the Proposed Similarity Measures

### 4.1. The Proposed Similarity Measures between SVNSs for Medical Diagnosis

We first give a numerical example about a medical diagnosis (adapted from Ye [19]) to illustrate the feasibility of the proposed new similarity measures *S*_1SVNS_^*∗*^ and *S*_2SVNS_^*∗*^ between SVNSs.

**Example 3 .**

Consider a medical diagnosis decision problem; suppose a set of diagnoses *Q*={*Q*_1_(viral  fever), *Q*_2_(malaria), *Q*_3_(typhoid), *Q*_4_(gastritis), *Q*_5_(stenocardia)} and a set of symptoms *S*={*S*_1_(fever), *S*_2_(headache), *S*_3_(stomach  pain), *S*_4_(cough), *S*_5_(chestpain)}. Assume a patient *P*_1_ has all the symptoms in the process of diagnosis, the SVNS evaluate information about *P*_1_ is

(13) $$\begin{array}{c} P_{1}\left(\text{Patient}\right)=\left\{\langle S_{1},0.8,0.2,0.1\rangle ,\langle S_{2},0.6,0.3,0.1\rangle ,\langle S_{3},0.2,0.1,0.8\rangle ,\langle S_{4},0.6,0.5,0.1\rangle ,\langle S_{5},0.1,0.4,0.6\rangle \right\}. \end{array}$$

The diagnosis information *Q*_*i*_(*i*=1,2,…, 5) with respect to symptoms *S*_*i*_(*i*=1,2,…, 5) also can be represented by the SVNSs, which is shown in Table 1.

By applying formulae (6) and (8), we can obtain the similarity measure values *S*_1SVNS_^*∗*^(*P*_1_, *Q*_*i*_) and *S*_2SVNS_^*∗*^(*P*_1_, *Q*_*i*_); the results are shown in Table 2.

From the above two similarity measures *S*_1SVNS_^*∗*^ and *S*_2SVNS_^*∗*^, we can conclude that the diagnoses of the patient *P*_1_ are all malaria (*Q*_2_). The proposed two similarity measures *S*_1SVNS_^*∗*^ and *S*_2SVNS_^*∗*^ produce the same results as Ye [19], which means the proposed similarity measures are feasible and effective.

### 4.2. The Proposed Similarity Measures between IVNSs for Medical Diagnosis

We know if the doctor examines the patient two or three times a day, then the interval values of multiple inspections for the patient are obtained. In this section, we will apply the proposed similarity measures *S*_1IVNS_^*∗*^ and *S*_2IVNS_^*∗*^ to medical diagnosis, the example is also adapted from Ye [19].

**Example 4 .**

Let us reconsider Example 3, assume a patient *P*_2_ has all the symptoms, which can be expressed by the following IVNS information.

(14) $$\begin{array}{c} P_{2}\left(\text{Patient}\right)=\left\{\langle S_{1},\left[0.3,0.5\right],\left[0.2,0.3\right],\left[0.4,0.5\right]\rangle ,\langle S_{2},\left[0.7,0.9\right],\left[0.1,0.2\right],\left[0.1,0.2\right]\rangle ,\langle S_{3},\left[0.4,0.6\right],\left[0.2,0.3\right],\left[0.3,0.4\right]\rangle ,\langle S_{4},\left[0.3,0.6\right],\left[0.1,0.3\right],\left[0.4,0.7\right]\rangle ,\langle S_{5},\left[0.5,0.8\right],\left[0.1,0.4\right],\left[0.1,0.3\right]\rangle \right\}. \end{array}$$

The same way as Example 3 in Ye [19], the diagnosis information of SVNSs *Q*_*i*_ with respect to symptoms *S*_*i*_(*i*=1,2, ⋯, 5) are transformed into IVNSs, which are shown in Table 3.

By applying formulae (10) and (12), we obtain the similarity measure values *S*_1IVNS_^*∗*^(*P*_2_, *Q*_*i*_) and *S*_2IVNS_^*∗*^(*P*_2_, *Q*_*i*_), the results are shown in Table 4.

From the two similarity measure values in Table 4, we can see that the patient *P*_2_ suffers from typhoid (*Q*_3_); the diagnosis results are the same as shown by Ye [19].

### 4.3. Comparative Analyses of Existing Similarity Measures

To illustrate the effectiveness of the proposed similarity measures for medical diagnosis, we will apply the existing similarity measures of SVNSs and IVNSs for comparative analyses.

At first, we introduce the existing similarity measures between SVNSs as follows:

Let *N*_1_={〈*x*_*i*_, *T*_*N*_1__(*x*_*i*_), *I*_*N*_1__(*x*_*i*_), *F*_*N*_1__(*x*_*i*_)〉|∣*x*_*i*_ ∈ *X*} and *N*_2_={*x*_*i*_, *T*_*N*_2__(*x*_*i*_), *I*_*N*_2__(*x*_*i*_), *F*_*N*_2__(*x*_*i*_)|∣*x*_*i*_ ∈ *X*} be two SVNSs in *X*={*x*_1_, *x*_2_,…, *x*_*n*_}, the existing similarity measures between *N*_1_ and *N*_2_ are defined as follows:

(1) Broumi et al. [23] proposed the similarity measure SM_SVNS_:
  (15) $$\begin{array}{c} {\text{SM}}_{\text{SVNS}}\left(N_{1},N_{2}\right)=1-D_{\text{SVNS}}\left(N_{1},N_{2}\right). \end{array}$$

(2) Şahin and Ahmet [24] proposed the similarity measure SD_SVNS_:
  (16) $$\begin{array}{c} {\text{SD}}_{\text{SVNS}}\left(N_{1},N_{2}\right)=\frac{1}{1+D_{\text{SVNS}}\left(N_{1},N_{2}\right)}. \end{array}$$
(3) Ye [19] proposed the improved cosine similarity measures SC_1SVNS_ and SC_2SVNS_:
  (17) $$\begin{array}{c} {\text{SC}}_{1\text{SVNS}}\left(N_{1},N_{2}\right)=\frac{1}{n}\overset{n}{\underset{i=1}{\sum }}\cos\left[\frac{\pi \cdot \max\left(\left|T_{N_{1}}\left(x_{i}\right)-T_{N_{2}}\left(x_{i}\right)\right|,\left|I_{N_{1}}\left(x_{i}\right)-I_{N_{2}}\left(x_{i}\right)\right|,\left|F_{N_{1}}\left(x_{i}\right)-F_{N_{2}}\left(x_{i}\right)\right|\right)}{2}\right], \end{array}$$
  (18) $$\begin{array}{c} {\text{SC}}_{2\text{SVNS}}\left(N_{1},N_{2}\right)=\frac{1}{n}\overset{n}{\underset{i=1}{\sum }}\cos\left[\frac{\pi \left(\left|T_{N_{1}}\left(x_{i}\right)-T_{N_{2}}\left(x_{i}\right)\right|+\left|I_{N_{1}}\left(x_{i}\right)-I_{N_{2}}\left(x_{i}\right)\right|+\left|F_{N_{1}}\left(x_{i}\right)-F_{N_{2}}\left(x_{i}\right)\right|\right)}{6}\right]. \end{array}$$
(4) Yang et al. [25] proposed the similarity measure SY_SVNS_(*N*_1_, *N*_2_):
  (19) $$\begin{array}{c} {\text{SY}}_{\text{SVNS}}\left(N_{1},N_{2}\right)=\frac{{\text{SC}}_{\text{SVNS}}\left(N_{1},N_{2}\right)}{{\text{SC}}_{\text{SVNS}}\left(N_{1},N_{2}\right)+D_{\text{SVNS}}\left(N_{1},N_{2}\right)}. \end{array}$$

**Example 5 ′.**

We apply formulae (5), (7), and (15)–(19) to calculate Example 5 again; the similarity measure values between *P*_1_ and *Q*_*i*_(*i*=1,2,…, 5) are shown in Table 5.

As we can see from Table 5, the patient *P*_1_ is still assigned to malaria (*Q*_2_), and the results are same as the proposed similarity measures in this paper, which means the proposed similarity measures are feasible and effective.

Next, we introduce the existing similarity measures between IVNSs as follows:

Let *N*_1_′={〈*x*_*i*_, [*T*_*N*_1_′_^*L*^(*x*_*i*_), *T*_*N*_1_′_^*U*^(*x*_*i*_)], [*I*_*N*_1_′_^*L*^(*x*_*i*_), *I*_*N*_1_′_^*U*^(*x*_*i*_)], [*F*_*N*_1_′_^*L*^(*x*_*i*_), *F*_*N*_1_′_^*U*^(*x*_*i*_)]〉|*x*_*i*_ ∈ *X*} and *N*_2_′={*x*_*i*_, [*T*_*N*_2_′_^*L*^(*x*_*i*_), *T*_*N*_2_′_^*U*^(*x*_*i*_)], [*I*_*N*_2_′_^*L*^(*x*_*i*_), *I*_*N*_2_′_^*U*^(*x*_*i*_)], [*F*_*N*_2_′_^*L*^(*x*_*i*_), *F*_*N*_2_′_^*U*^(*x*_*i*_)]|*x*_*i*_ ∈ *X*} be two IVNSs in *X*={*x*_1_, *x*_2_,…, *x*_*n*_}, the existing similarity measures between *N*_1_′ and *N*_2_′ are defined as follows:

(1) Broumi et al. [23] proposed the similarity measure SM_IVNS_:
  (20) $$\begin{array}{c} {\text{SM}}_{\text{IVNS}}\left(N_{1}^{\prime },N_{2}^{\prime }\right)=1-D_{\text{IVNS}}\left(N_{1}^{\prime },N_{2}^{\prime }\right). \end{array}$$
(2) Şahin and Ahmet [24] proposed the similarity measure SD_IVNS_:
  (21) $$\begin{array}{c} {\text{SD}}_{\text{IVNS}}\left(N_{1}^{\prime },N_{2}^{\prime }\right)=\frac{1}{1+D_{\text{IVNS}}\left(N_{1}^{\prime },N_{2}^{\prime }\right)}. \end{array}$$
(3) Broumi and Smarandache [22] proposed the improved cosine similarity measures SC_1SVNS_ and SC_2SVNS_:
  (22) $$\begin{array}{c} {\text{SC}}_{1\text{IVNS}}\left(N_{1}^{\prime },N_{2}^{\prime }\right)=\frac{1}{n}\overset{n}{\underset{i=1}{\sum }}\cos\frac{\pi }{4}\left[\max\left(\left|T_{N_{1}^{\prime }}^{L}\left(x_{i}\right)-T_{N_{2}^{\prime }}^{L}\left(x_{i}\right)\right|,\left|I_{N_{1}^{\prime }}^{L}\left(x_{i}\right)-I_{N_{2}^{\prime }}^{L}\left(x_{i}\right)\right|,\left|F_{N_{1}^{\prime }}^{L}\left(x_{i}\right)-F_{N_{2}^{\prime }}^{L}\left(x_{i}\right)\right|\right)\right.+\left.\max\left(\left|T_{N_{1}^{\prime }}^{U}\left(x_{i}\right)-T_{N_{2}^{\prime }}^{U}\left(x_{i}\right)\right|,\left|I_{N_{1}^{\prime }}^{U}\left(x_{i}\right)-I_{N_{2}^{\prime }}^{U}\left(x_{i}\right)\right|,\left|F_{N_{1}^{\prime }}^{U}\left(x_{i}\right)-F_{N_{2}^{\prime }}^{U}\left(x_{i}\right)\right|\right)\right], \end{array}$$
  (23) $$\begin{array}{c} {\text{SC}}_{2\text{IVNS}}\left(N_{1}^{\prime },N_{2}^{\prime }\right)=\frac{1}{n}\overset{n}{\underset{i=1}{\sum }}\cos\frac{\pi }{12}\left(\left|T_{N_{1}^{\prime }}^{L}\left(x_{i}\right)-T_{N_{2}^{\prime }}^{L}\left(x_{i}\right)\right|+\left|I_{N_{1}^{\prime }}^{L}\left(x_{i}\right)-I_{N_{2}^{\prime }}^{L}\left(x_{i}\right)\right|+\left|F_{N_{1}^{\prime }}^{L}\left(x_{i}\right)-F_{N_{2}^{\prime }}^{L}\left(x_{i}\right)\right|\right.+\left.\left|T_{N_{1}^{\prime }}^{U}\left(x_{i}\right)-T_{N_{2}^{\prime }}^{U}\left(x_{i}\right)\right|+\left|I_{N_{1}^{\prime }}^{U}\left(x_{i}\right)-I_{N_{2}^{\prime }}^{U}\left(x_{i}\right)\right|+\left|F_{N_{1}^{\prime }}^{U}\left(x_{i}\right)-F_{N_{2}^{\prime }}^{U}\left(x_{i}\right)\right|\right). \end{array}$$
(4) Yang et al. [25] proposed the similarity measure SY_SVNS_(*N*_1_′, *N*_2_′):
  (24) $$\begin{array}{c} {\text{SY}}_{\text{IVNS}}\left(N_{1}^{\prime },N_{2}^{\prime }\right)=\frac{{\text{SC}}_{\text{IVNS}}\left(N_{1}^{\prime },N_{2}^{\prime }\right)}{{\text{SC}}_{\text{IVNS}}\left(N_{1}^{\prime },N_{2}^{\prime }\right)+D_{\text{IVNS}}\left(N_{1}^{\prime },N_{2}^{\prime }\right)}. \end{array}$$

***Example 4′*.**

Applying formulae (9), (11), and (20)–(24) to calculate Example 6 again, the similarity measure values between *P*_2_ and *Q*_*i*_(*i*=1,2,…, 5) are shown in Table 6.

The results of Table 6 show that the patient *P*_2_ should be assigned to typhoid (*Q*_3_), they are same as the proposed similarity measures *S*_1IVNS_^*∗*^ and *S*_2IVNS_^*∗*^ in the paper, which means the proposed methods are feasible and effective.

The proposed similarity measures in the paper have some advantages in solving multiple criteria decision-making problems. They are constructed based on the existing similarity measures and Euclidean distance, which not only satisfy the axiom of the similarity measure but also consider the similarity measure from the points of view of algebra and geometry. Furthermore, they can be applied more widely in the field of decision-making problems.

## 5. Conclusions

The similarity measure is widely used in multiple criteria decision-making problems. This paper proposed a new method to construct the similarity measures combining the existing cosine similarity measure and the Euclidean distance measure of SVNSs and IVNSs, respectively, which are based on the above existing similarity measures and the Euclidean distance measure. And, the similarity measures are proposed not only from the points of view of algebra and geometry but also satisfy the axiom of the similarity measure. Furthermore, we apply the proposed similarity measures to medical diagnosis decision problems, and the numerical example is used to illustrate the feasibility and effectiveness of the proposed similarity measure, which are then compared to other existing similarity measures. In future research, we will focus on studying the similarity measure between linguistic neutrosophic set and the application of the proposed similarity measures of neutrosophic sets, such as pattern recognition, supplier selection, and so on.

## Figures and Tables

**Table 1:** The relation between the diagnosis and the symptom for SVNS decision information.

	*S*_1_	*S*_2_	*S*_3_	*S*_4_	*S*_5_
*Q*_1_	<0.4, 0.6, 0.0>	<0.3, 0.2, 0.5>	<0.1, 0.3, 0.7>	<0.4, 0.3, 0.3>	<0.1, 0.2, 0.7>
*Q*_2_	<0.7, 0.3, 0.0>	<0.2, 0.2, 0.6>	<0.0, 0.1, 0.9>	<0.7, 0.3, 0.0>	<0.1, 0.1, 0.8>
*Q*_3_	<0.3, 0.4, 0.3>	<0.6, 0.3, 0.1>	<0.2, 0.1, 0.7>	<0.2, 0.2, 0.6>	<0.1, 0.0, 0.9>
*Q*_4_	<0.1, 0.2, 0.7>	<0.2, 0.4, 0.4>	<0.8, 0.2, 0.0>	<0.2, 0.1, 0.7>	<0.2, 0.1, 0.7>
*Q*_5_	<0.1, 0.1, 0.8>	<0.0, 0.2, 0.8>	<0.2, 0.0, 0.8>	<0.2, 0.0, 0.8>	<0.8, 0.1, 0.1>

We can calculate the similarity measures *S*_1SVNS_^*∗*^(*P*_1_, *Q*_*i*_) and *S*_2SVNS_^*∗*^(*P*_1_, *Q*_*i*_)(*i*=1,2,…, 5), and then the diagnoses of the patient *P*_1_ can be classified by $R_{j}=\arg\underset{1\leq i\leq 5}{\max}\left\{S_{j\text{SVNS}}^{*}\left(P_{1},Q_{i}\right)\right\}\left(j=1,2\right)$.

**Table 2:** The similarity measures between *P*_1_ and *Q*_*i*_.

	*Q*_1_	*Q*_2_	*Q*_3_	*Q*_4_	*Q*_5_
*S*_1SVNS_^*∗*^(*P*_1_, *Q*_*i*_)	0.6663	**0.7188**	0.5387	0.4594	0.4336
*S*_2SVNS_^*∗*^(*P*_1_, *Q*_*i*_)	0.8223	**0.8378**	0.6377	0.5500	0.4881

**Table 3:** The relation between the diagnosis and the symptom for IVNS decision information.

	*S*_1_	*S*_2_	*S*_3_	*S*_4_	*S*_5_
*Q*_1_	<[0.4, 0.4], [0.6, 0.6], [0.0, 0.0]>	<[0.3, 0.3], [0.2, 0.2], [0.5, 0.5]>	<[0.1, 0.1], [0.3, 0.3], [0.7, 0.7]>	<[0.4, 0.4], [0.3, 0.3], [0.3, 0.3]>	<[0.1, 0.1], [0.2, 0.2], [0.7, 0.7]>
*Q*_2_	<[0.7, 0.7], [0.3, 0.3], [0.0, 0.0]>	<[0.2, 0.2], [0.2, 0.2], [0.6, 0.6]>	<[0.0, 0.0], [0.1, 0.1], [0.9, 0.9]>	<[0.7, 0.7], [0.3, 0.3], [0.0, 0.0]>	<[0.1, 0.1], [0.1, 0.1], [0.8, 0.8]>
*Q*_3_	<[0.3, 0.3], [0.4, 0.4], [0.3, 0.3]>	<[0.6, 0.6], [0.3, 0.3], [0.1, 0.1]>	<[0.2, 0.2], [0.1, 0.1], [0.7, 0.7]>	<[0.2, 0.2], [0.2, 0.2], [0.6, 0.6]>	<[0.1, 0.1], [0.0, 0.0], [0.9, 0.9]>
*Q*_4_	<[0.1, 0.1], [0.2, 0.2], [0.7, 0.7]>	<[0.2, 0.2], [0.4, 0.4], [0.4, 0.4]>	<[0.8, 0.8], [0.2, 0.2], [0.0, 0.0]>	<[0.2, 0.2], [0.1, 0.1], [0.7, 0.7]>	<[0.2, 0.2], [0.1, 0.1], [0.7, 0.7]>
*Q*_5_	<[0.1, 0.1], [0.1, 0.1], [0.8, 0.8]>	<[0.0, 0.0], [0.2, 0.2], [0.8, 0.8]>	<[0.2, 0.2], [0.0, 0.0], [0.8, 0.8]>	<[0.2, 0.2], [0.0, 0.0], [0.8, 0.8]>	<[0.8, 0.8], [0.1, 0.1], [0.1, 0.1]>

We can calculate the similarity measures *S*_1IVNS_^*∗*^(*P*_2_, *Q*_*i*_) and *S*_2IVNS_^*∗*^(*P*_2_, *Q*_*i*_)(*i*=1,2, ⋯, 5), and the diagnosis of the patient *P*_2_ can be classified by $R_{j}=\arg\underset{1\leq i\leq 5}{\max}\left\{S_{j\text{IVNS}}^{*}\left(P_{2},Q_{i}\right)\right\}\left(j=1,2\right)$.

**Table 4:** The similarity measures between *P*_2_ and *Q*_*i*_.

	*Q*_1_	*Q*_2_	*Q*_3_	*Q*_4_	*Q*_5_
*S*_1IVNS_^*∗*^(*P*_2_, *Q*_*i*_)	0.5783	0.4610	**0.6273**	0.5772	0.5401
*S*_2IVNS_^*∗*^(*P*_2_, *Q*_*i*_)	0.6804	0.5729	**0.7503**	0.7061	0.6734

**Table 5:** Comparisons of different similarity measure between SVNSs.

	*Q*_1_	*Q*_2_	*Q*_3_	*Q*_4_	*Q*_5_
SM_SVNS_ [23]	0.7941	**0.8094**	0.4568	0.5851	0.5517
SD_SVNS_ [24]	0.8293	**0.8399**	0.6480	0.7067	0.6905
SC_1SVNS_ [22]	0.8942	**0.8976**	0.8422	0.6102	0.5607
SC_2SVNS_ [22]	0.9443	**0.9571**	0.9264	0.8214	0.7650
SY_SVNS_ [25]	0.8128	**0.8248**	0.6079	0.5953	0.5557
*S*_1SVNS_ [15]	0.5385	**0.6282**	0.6206	0.3336	0.3154
*S*_2SVNS_ [18]	0.8505	**0.8661**	0.8185	0.5148	0.4244

**Table 6:** Comparisons of different similarity measures between IVNSs.

	*Q*_1_	*Q*_2_	*Q*_3_	*Q*_4_	*Q*_5_
SM_IVNS_ [23]	0.6833	0.5844	**0.7265**	0.6923	0.6596
SD_IVNS_ [24]	0.7595	0.7064	**0.7852**	0.7647	0.7460
SC_1IVNS_ [22]	0.7283	0.6079	**0.7915**	0.7380	0.7157
SC_2IVNS_ [22]	0.8941	0.8459	**0.9086**	0.9056	0.8797
SY_IVNS_ [25]	0.6969	0.5939	**0.8429**	0.7057	0.6777
S_1IVNS_ [22]	0.4733	0.3376	**0.5209**	0.4620	0.4205
S_2IVNS_ [21]	0.6775	0.5613	**0.7741**	0.7198	0.6872

## Data Availability

The data used to support the findings of this study are included within the article.

## References

[1] Zadeh L. A. (1965). Fuzzy sets. *Information and Control*.

[2] De S. K., Biswas R., Roy A. R. (1998). Multicriteria decision making using intuitionistic fuzzy set theory. *Journal of Fuzzy Mathematics*.

[3] Phuong N. H., Thang V. V. (2000). Case based reasoning for medical diagnosis using fuzzy set theory. *International Journal of Biomedical Soft Computing and Human Sciences*.

[4] Tobias O. J., Seara R. (2002). Image segmentation by histogram thresholding using fuzzy sets. *IEEE Transactions on Image Processing*.

[5] Zadeh L. A. (1974). The concept of a linguistic variable and its application to approximate reasoning. *Information Science*.

[6] Atanassov K. T., Rangasamy P. (1986). Intuitionistic fuzzy sets. *Fuzzy Sets and Systems*.

[7] Wang H., Smarandache F., Zhang Y. (2013). Single valued neutrosophic sets. *Review of the Air Force Academy*.

[8] Wang H. (2005). Interval neutrosophic sets and logic: theory and applications in computing. *Computer Science*.

[9] Ye J. (2014). Multiple attribute group decision-making method with completely unknown weights based on similarity measures under single valued neutrosophic environment. *Journal of Intelligent and Fuzzy Systems*.

[10] Arockiarani I., Sumathi I. R. (2014). Some results on interval valued Fuzzy neutrosophic soft set. *International Journal of Innovative Research and Studies*.

[11] Mukherjee A., Sarkar S. (2014). Several similarity measures of neutrosophic soft sets and its application in real life problems. *Annals of Pure and Applied Mathematics*.

[12] Ye J. (2014). Similarity measures between interval neutrosophic sets and their applications in multicriteria decision-making. *Journal of Intelligent and Fuzzy Systems*.

[13] Beg (2009). Similarity measures for fuzzy sets. *Applied and Computational Mathematics*.

[14] Song Y., Wang X., Zhang H. (2015). A distance measure between intuitionistic fuzzy belief functions. *Knowledge-Based Systems*.

[15] Majumdar P., Samanta S. K. (2013). On similarity and entropy of neutrosophic sets. *Journal of Intelligent and Fuzzy Systems*.

[16] Ye J. (2016). Multicriteria decision-making method based on cosine similarity measures between interval- valued fuzzy sets with risk preference. *Economic Computation and Economic Cybernetics Studies and Research*.

[17] Ye J. (2011). Cosine similarity measures for intuitionistic fuzzy sets and their applications. *Mathematical and Computer Modelling*.

[18] Ye J. (2014). Vector similarity measures of simplified neutrosophic sets and their application in multicriteria decision making. *International Journal of Fuzzy Systems*.

[19] Ye J. (2015). Improved cosine similarity measures of simplified neutrosophic sets for medical diagnoses. *Artificial Intelligence In Medicine*.

[20] Liu D., Chen X., Peng D. (2017). Interval-valued intuitionistic fuzzy ordered weighted cosine similarity measure and its application in investment decision-making. *Complexity*.

[21] Liu D., Chen X., Peng D. (2018). The intuitionistic fuzzy linguistic cosine similarity measure and its application in pattern recognition. *Complexity*.

[22] Broumi S., Smarandache F. New distance and similarity measures of interval neutrosophic sets.

[23] Broumi S., Smarandache F. (2013). Several similarity measures of neutrosophic sets. *Neutrosophic Sets and Systems*.

[24] Şahin R., Ahmet K. (2014). *On Similarity and Entropy of Neutrosophic Soft Sets*.

[25] Yang Y., Zhang R., Guo J. (2017). A multi-attribute decision-making approach based on hesitant neutrosophic sets. *Fuzzy Systems and Mathematics*.

